# Electrochemical sensor based on the synergy between Cucurbit[8]uril and 2D-MoS_2_ for enhanced melatonin quantification

**DOI:** 10.1038/s41598-023-37401-9

**Published:** 2023-06-27

**Authors:** Rut Martínez-Moro, María del Pozo, Luis Vázquez, José A. Martín-Gago, María Dolores Petit-Domínguez, Elena Casero, Carmen Quintana

**Affiliations:** 1 Departamento de Química Analítica y Análisis Instrumental. Facultad de Ciencias, c/ Francisco Tomás y Valiente, Nº7. Campus de Excelencia de la Universidad Autónoma de Madrid, 28049 Madrid, Spain; 2 Instituto de Ciencia de Materiales de Madrid (CSIC), c/ Sor Juana Inés de la Cruz Nº3. Campus de Excelencia de la Universidad Autónoma de Madrid, 28049 Madrid, Spain

**Keywords:** Sensors, Bioanalytical chemistry, Electrochemistry, Supramolecular chemistry, Two-dimensional materials

## Abstract

We present the development of an electrochemical sensor towards melatonin determination based on the synergistic effect between MoS_2_ nanosheets and cucurbit[8]uril. For the sensor construction cucurbit[8]uril suspensions were prepared in water, and MoS_2_ nanosheets were obtained by liquid exfoliation in ethanol:water. The sensing platform was topographically characterized by Atomic Force Microscopy. Electrochemical Impedance Spectroscopy experiments allowed us to study the charge transfer process during melatonin oxidation. Moreover, stoichiometry of the resulting complex has also been determined. After the optimization of the sensor construction and the experimental variables involved in the Differential Pulse Voltammetric response of melatonin, detection limit of 3.80 × 10^−7^ M, relative errors minor than 3.8% and relative standard deviation lower than 4.4% were obtained. The proposed sensor has been successfully applied to melatonin determination in pharmaceutical and biological samples as human urine and serum, with very good recoveries ranging from 90 to 102%.

## Introduction

Determination of biological interest molecules is of great importance nowadays. One of them is melatonin (MEL), N-acetyl-5methoxy tryptamine, an endogenous hormone derived from serotonin synthesized by many living organisms including plants. MEL main functions are the regulation of circadian rhythms and blood pressure, hence its popular use to treat sleep disorders and jet-lag effects. It counteracts the effects of free radicals and acts as an antioxidant and anti-inflammatory agent. According to recent studies, abnormal MEL levels might be associated to an increased risk of developing breast cancer. Also noteworthy, the use of MEL as a complement in neurodegenerative diseases such as Parkinson, Alzheimer’s disease and epilepsy to improve the quality of life of patients have shown promising results^1–3^.

Previously mentioned data made evident the importance of accurate MEL determination both in pharmaceutical and biological samples. To date, several techniques are used for MEL detection and quantification including spectrofluorimetry^4^, capillary electrophoresis^5^ and chromatography coupled to different detectors as diode- array, fluorescence, or mass spectrometry^6–8^, to name a few. However, analysis of MEL applying electrochemical techniques is less popular even though they present several advantages when compared to others, such as their low-cost, ease of handling, absence of sample pre-treatment usually, and almost immediately data output. Electrochemical analysis of MEL is based on molecule oxidation on different surfaces, mainly carbon electrodes^9,10^, which can be modified using different nanomaterials^11–16^. These electrode surface modifiers possess not only individual properties that might improve, for example, the electrical conductivity, but also synergistic properties when combined.

Transition metal dichalcogenides (TMDs) nanosheets present unique and improved properties, hence the importance they have gained. These 2D graphene like materials can be obtained from its bulk materials applying top-down methodologies (liquid exfoliation and chemical exfoliation to name some) and by bottom-up strategies starting from its atomic components. Their structure consists of vertically stacked X-M-X interlayers, being M a transition metal atom and X a chalcogen atom, with MX_2_ stoichiometry in a sandwich like structure^17–20^. TMDs nanosheets have attracted much interest in electrochemical applications for developing new analytical sensors. Several studies have observed a synergistic effect between TMDs nanosheets and other nanomaterials (i.e. gold nanoparticles, diamond nanoparticles)^21–24^ or organic macromolecules such as enzymes^25^. Among the different TMDs, MoS_2_ nanosheets are the ones that have attracted most research efforts in electrochemical applications.

Researchers have found a great improvement in selectivity of electrochemical sensors through host—guest interactions by using supramolecular receptors as electrode surface modifiers. Among them, although cyclodextrins have been the most studied ones, cucurbit[n]uril (CB[n]) has become an important family of macrocycles that has attracted great interest over the last decade^26,27^. Formed by glycoluril units bonded by methylene bridges in a pumpkin-like shape, these compounds present a hydrophobic cavity which can be accessed via two symmetrical portals with “n” carbonyl groups. This structure allows CB[n] to form inclusion complexes with a wide variety of molecules not only based on their size but also on their electronic properties. Hydrophobic or relatively hydrophobic guests of an appropriate size can be included in the non-polar cavity, while the carbonyl groups present at the portals allow electrostatic or ion–dipole interactions with positively charged analytes. In addition, this negative charge density allows the stabilization of inclusion complexes in the case of cationic guests^26–30^.

Regarding host–guest interactions between MEL and molecular receptors, MEL forms an inclusion complex in the presence of β-cyclodextrin which has been studied by spectroscopic techniques increasing MEL solubility in water^31^.

In a previous fundamental work, through a combination of experimental techniques, such as Nuclear Magnetic Resonance spectroscopy, and theoretical methods based on Density Functional Theory (DFT) and Molecular Dynamics (MD) calculations, we addressed the study of the adsorption of CB[8] on different 2D surfaces. We concluded that depending on the CB[8] adsorption geometry on the TMD surface either one (on graphene) or both (on MoS_2_) cavity portals are exposed to the solution containing the potential guest molecule^32^. This evidence together with the reported synergistic effect appearing when using TMDs nanosheets, and the excellent CB[8] properties as molecular selector through host–guest interactions, motivate us to focus on the development and fully characterization of an electrochemical sensor based on MoS_2_ and CB[8] for MEL determination in real samples.

## Results and discussion

### Electrochemical sensor design

The remarkable performance as molecular selectors demonstrated by CB[n]s makes them excellent compounds to increase the selectivity of electrochemical sensors^27,28^. On the other hand, the synergistic effect produced by the combination of 2D-TMDs with different nanomaterials has been previously reported and employed for the development of electrochemical sensors with improved properties. Based on these facts we addressed the development of an electrochemical dispositive including 2D-MoS_2_ together with the CB[8] as modifiers of a GC electrode towards MEL determination. The validity of this approach requires demonstrating that the presence of both materials on the surface of the electrode is necessary to achieve these improvements.

**Figure 1:**
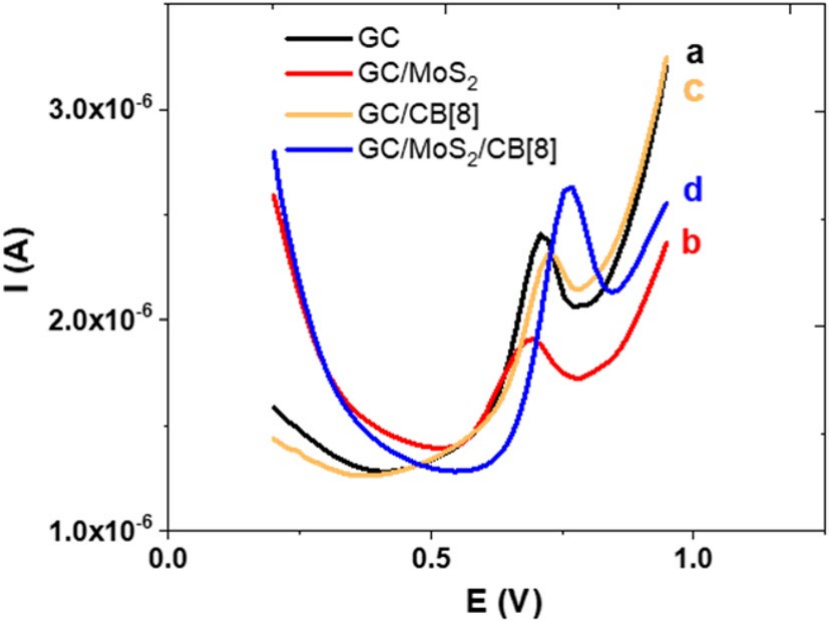
DPV response of (**a**) bare GC, (**b**) GC/MoS_2_, (**c**) GC/CB[8] and (**d**) GC/MoS_2_/CB[8] in 0.1 M pH = 7.0 phosphate buffer solution containing 1 × 10^–5^ M of MEL. DPV conditions: scan rate 30 mVs^−1^, pulse amplitude 60 mV and step potential 15 mV.

Figure 1 shows the differential pulse voltammograms recorded with different sensors: the GC/MoS_2_ system, the GC/CB[8] system and the complete dispositive namely GC/MoS_2_/CB[8]. In addition, the response obtained with a non-modified GC electrode is also included for comparison. In this later case (voltammogram a), the MEL oxidation recorded at c.a. 0.7 V is in good agreement with that described for MEL oxidation on this electrode surface^10^. When the electrode is modified just with the 2D-MoS_2_ (GC/MoS_2_ system, voltammogram b), the semiconductor character of the TMD results in a decrease of the peak current, an electrochemical behaviour previously reported for other analytes^22^. The response observed with the GC/CB[8] system (voltammogram c), agrees with the expected electrochemical response for a supramolecular complex, i.e., lower peak current and even a slight potential peak shift to higher values. The response of MEL with the sensor resulting of the modification with both nanomaterials is depicted in voltammogram d. In this case, the inclusion of MEL in the receptor cavity can be concluded from the peak potential shift. However, it is worth to notice that a great increase current is produced in contrast with the decrease expected due to the complex formation. Whereas such current increase has been previously described in hybrid bilayer systems combining TMDs nanosheets and different 0D nanomaterials as gold nanoparticles^33^ or diamond nanoparticles^21^, this is the first time that it is observed when combining only TMDs nanosheets with supramolecular hosts. These results evidence the benefits of combining both materials, a sensor with improved response in terms of sensitivity derived from the synergistic effect between TMDs and CB[8] but also in terms of selectivity from the macrocycle properties of the latter as molecular selector.

Moreover, DPV experiments allowed us to stablish the stoichiometry of the complex following the molar ratio procedure. To this end, we recorded the voltammograms corresponding to 1 × 10^–5^ M MEL without CB[8] and with increasing macrocycle amounts employing the GC electrode. The results, depicted in Fig. S1 let us to conclude that one MEL molecule is included in the receptor cavity in a 1:1 ratio.

### Optimization of the sensor design

**Figure 2:**
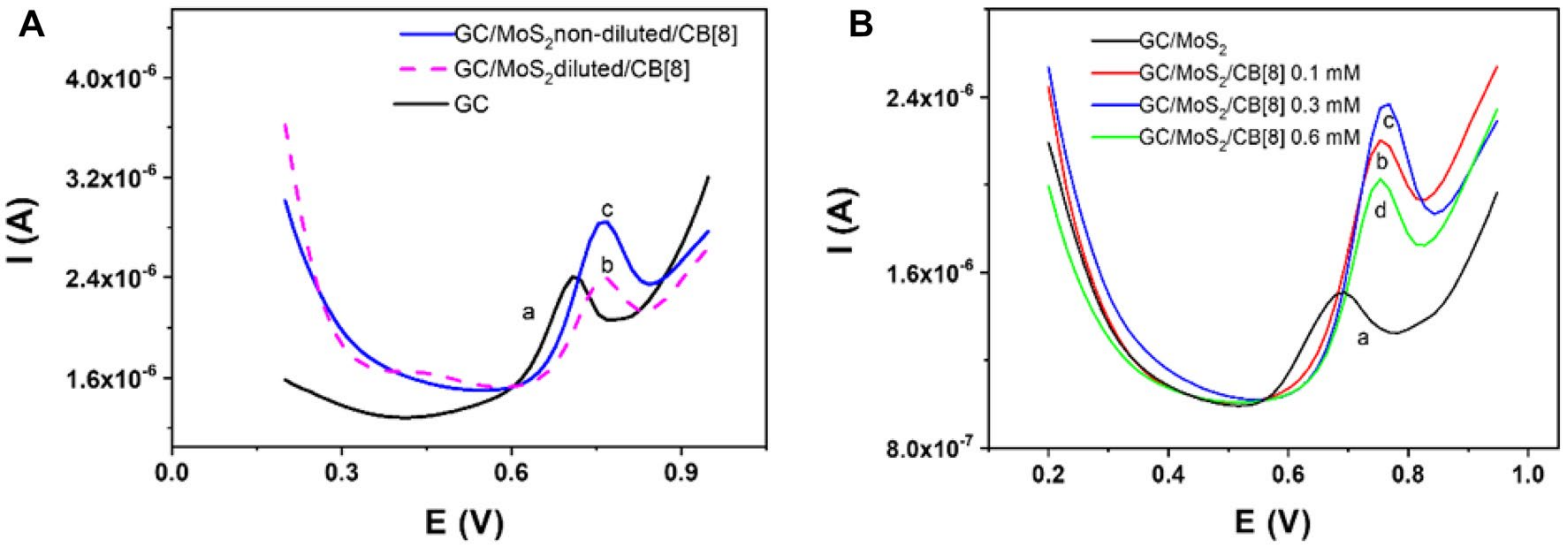
(**A**) DPV response of 10^–5^ M MEL in a GC electrode (a) in a GC/MoS_2_/CB[8] system prepared with 1:1 (v/v) diluted MoS_2_ suspensions (b, dashed line) and with non-diluted MoS_2_ suspensions (c). (**B**) DPV response of 1 × 10^–5^ M MEL with GC/MoS_2_ (non-diluted) without CB[8] (a), GC/MoS_2_/CB[8] electrode containing non-diluted MoS_2_ and different CB[8] amounts: 0.1 mM CB[8] (b), 0.3 mM CB[8] (c), 0.6 mM CB[8] (d).

Once the synergistic effect of combining 2D-MoS_2_ (7.5 mg/mL) and CB[8] is demonstrated, we address the optimization of the dispositive design in terms of the amounts of MoS_2_ and macrocyclic used for the electrode modification. Figure 2A shows the DPV response of MEL with two different GC/MoS_2_/CB[8] sensors prepared with a 1:1 (v/v) diluted MoS_2_ suspension (voltammogram b) and with a non-diluted MoS_2_ suspension (voltammogram c) together with the response obtained with the bare GC electrode (voltammogram a) for comparison. It is clear that the synergistic effect resulting from the combination TMD/CB[8] is produced when using the synthetized MoS_2_ suspensions without dilution, probably as a consequence of the increase in the available surface generated by the use of the TMD.

Next, the influence of the macromolecular receptor amount was evaluated, and the results are depicted in Fig. 2B. As observed, an increase of the CB[8] amount on the electrode surface leads to a higher peak current up to 0.3 mM CB[8] concentration. The decrease observed for 0.6 mM concentration could be explained by the extremely low CB[8] solubility resulting in less homogeneous suspensions. Moreover, at this CB[8] concentration, the reproducibility in the modification process and in the performance of the device, was poorer. As a result of these experiments, the sensor was prepared with the non-diluted MoS_2_ initial synthetized suspension and maintaining the receptor concentration at 0.3 mM.

### AFM characterization

In the following, we will describe the main results obtained from the AFM analysis for different samples according to the sensor construction: Si/CB[8], Si/MoS_2_ and Si/MoS_2_/CB[8].

#### Si/CB[8]

**Figure 3:**
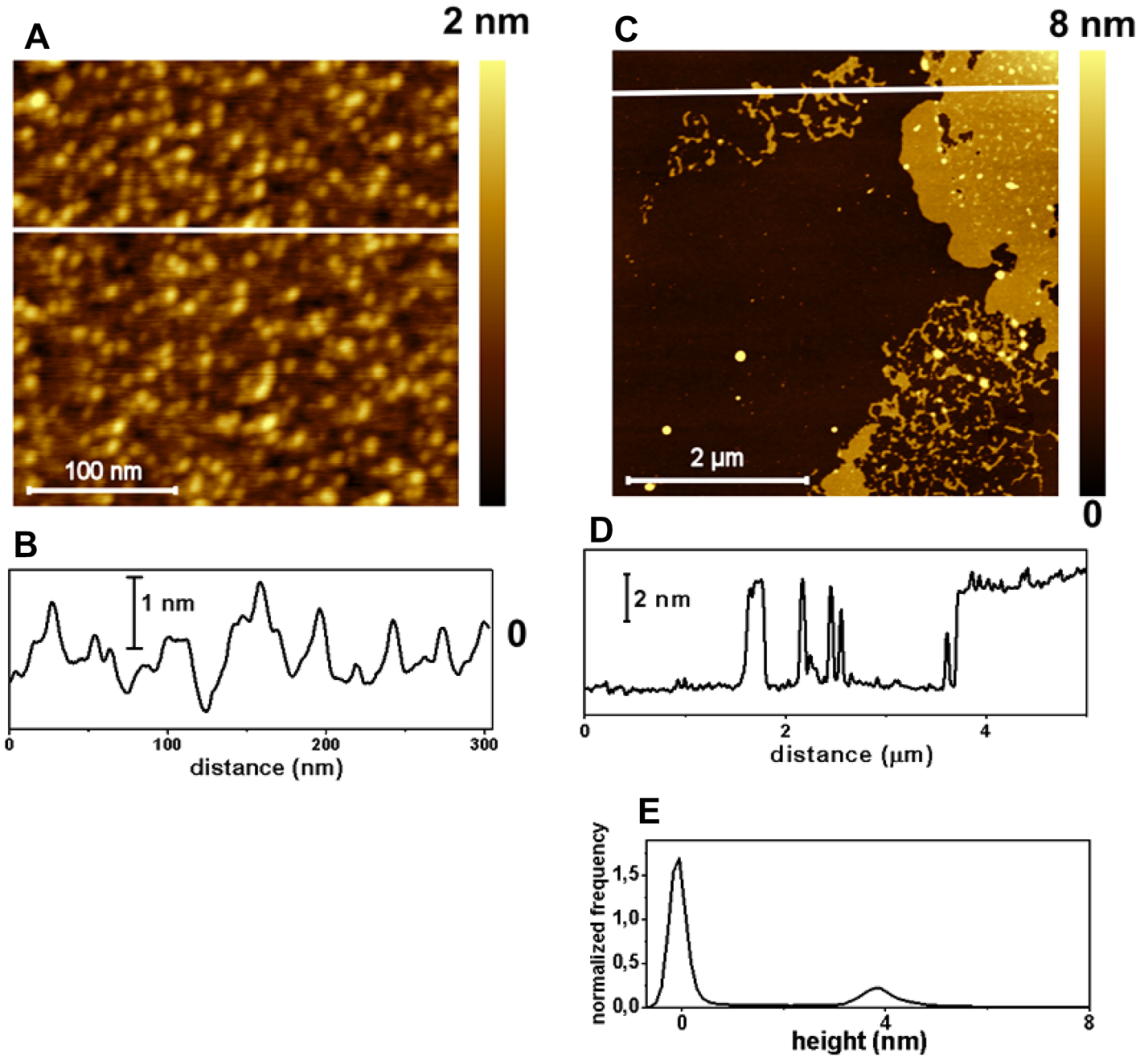
(**A**) Top view AFM image of the Si/CB[8] sample. (**B**) Surface profile along the line drawn in (**A**). (**C**) AFM image of the Si/MoS_2_ sample. (**D**) Surface profile along the line drawn in (**C**). (**E**) Height distribution of (**C**). The highest peak corresponds to the substrate areas whereas the smaller one is due to the surface of the flakes.

The surface morphology of this sample was quite homogeneous as can be seen in Fig. 3A. Here, globular structures are found over the surface. In the surface profile (Fig. 3B) along the line depicted in Fig. 3A structures around 1–2 nm high are observed. It should be noted that the height of 1–2 nm is close to the CB[8] size with an equatorial width of 1.75 nm, and height of 0.91 nm, including van der Waals radii^26^. In contrast, the corresponding width, measured at half height, yields values in the 8–10 nm. This large value can be explained by the convolution effects due to the tip size (nominal tip radius of 8 nm). Thus, one structure with a size around 1–2 nm can be visualized by AFM with an enlarged lateral size, compatible with the 10 nm value measured, whereas its height will be close to the expected one, provided that the tip load is weak. Therefore, in principle, these 1–2 nm high globular structures could be consistent with single CB[8] molecules. In addition, this morphology is consistent with previous results^34^. A detailed inspection of the background in the image allows to detect up to two depth levels. Furthermore, in the top one, tiny globular structures can be spotted. This fact suggests that a sort of CB[8] multilayer deposit is formed. This fact is confirmed when a similar sample, where the CB[8] concentration is the tenth of the one used in this measurement, is studied since, in this case, a continuous layer is still visible (not shown).

#### Si/MoS_2_ sample

The morphology is characterized by the presence of flakes (Fig. 3C). These flakes have an average thickness close to 4 nm. This can be observed in Fig. 3D showing the profile along the line drawn in Fig. 3C. At the right, the sharp step at the flake is clearly seen. In the middle of the profile tiny structures can be seen with a similar thickness that could correspond to flake fragments, which are produced during the preparation method. A statistical analysis of the thickness of the flakes is obtained from the height distribution of the AFM image where the x-axis is the pixel height (Fig. 3E). The peak around zero in the x-axis corresponds to the substrate whereas the second one around 4 comes from the surface of the flakes (the former is higher because the substrate area is larger than the flake one). In Fig. 3C and D are detected small structures on top of the flake ones. They cover roughly the 15% of the flake surface. They could also correspond to tiny products of the preparation method left after the drop drying.

#### Si/MoS_2_/CB[8] sample

**Figure 4:**
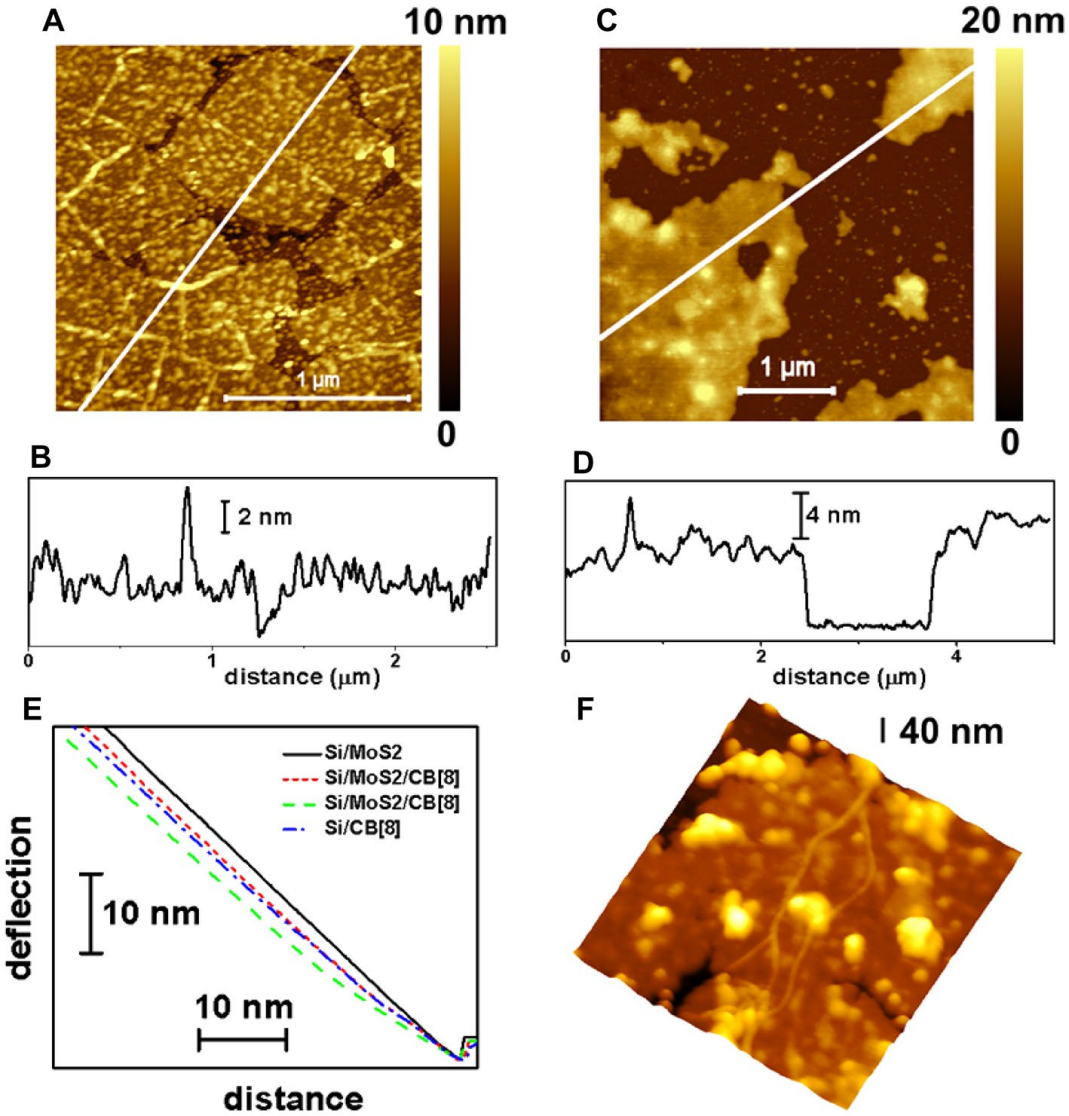
AFM data of the Si/MoS_2_/CB[8]. (**A**) Large top view AFM image. (**B**) Surface profile along the line drawn in (**A**). (**C**) AFM image obtained on other location. (**D**) Surface profile along the line drawn in (**C**). (**E**) Force curves obtained with the same cantilever on the structures indicated in the legend. (**F**) 900 × 900 nm^2^ 3D AFM image taken on top of a MoS_2_ flake. Note the fiber structure running from the top to the bottom of the image.

A typical morphology of this system is shown in Fig. 4A where the MoS_2_ flakes are covered by multiple nanostructures. The line drawn in Fig. 4A crosses the main flake. Its thickness can be measured in the corresponding surface profile (Fig. 4B), with a value close to 4 nm, as well as the height of the tiny isolated nanostructures that results to be in the 1–2 nm range, although there are also larger aggregates. These nanostructures cover around the 66% of the flake surfaces. Noticeably, fiber structures are also observed in the image. This fiber-like structures have been reported before for a CB[7] gel^35^. In our case, the fiber dimensions suggest a sort of fibril aggregation as already reported by Hwang et al.^35^. In contrast, in other location of the sample, morphology as that shown in Fig. 4C is found. Here, the MoS_2_ flakes are evident but this time they are fully covered by a continuous layer of material, in contrast with the surrounding substrate surface in which isolated deposited structures are imaged. The surface profile along the line drawn in Fig. 4C is displayed in Fig. 4D. Now, the thickness of the flakes is clearly higher than 4 nm, with values from 6 up to 11 nm, depending on the location. In fact, the surface is rougher than that of a bare MoS_2_ flake.

In order to further assess the nature of the deposits on top of the flakes, we performed force curve measurements on Si/MoS_2_ and Si/MoS_2_/CB[8] samples. The results are displayed in Fig. 4E. In the curves, the x-axis represents the distance travelled by the tip towards the surface (from right to left). At the right, there is a horizontal region indicating that the tip is not in contact with the surface, yet. Then, there is a decrease in the deflection due to the attractive Van der Waals forces between tip and sample. From the minimum deflection point to the left in the x-axis, the tip is in contact with the surface and repulsive forces operate leading to an increase in the positive cantilever deflection. The curve taken on the MoS_2_ flake shows a steep slope, in fact close to that obtained on the hard reference sapphire sample (not shown), which indicates its rigid and hard character. As a reference, we obtained curves also on the Si-CB[8] sample (dot-dashed curve). This curve has initially a curved shape with a smaller slope before reaching a similar slope than that observed on the MoS_2_ flake. This initial curved region corresponds to the sampling of the soft CB[8] structure, and the straight sloped region is attained once the tip has gone through the soft layer. The lateral shift of the curve with respect to that one measured on MoS_2_, at a fixed and high y-axis value, gives a value of the thickness of the CB[8] deposit that results to be 3.5–4 nm. In addition, we measured force curves on flakes of the Si/MoS_2_/CB[8] sample, the short dashed curve taken on a flake as those of Fig. 4A, and the dashed one obtained on a flake as that of Fig. 4C. Both display an initial curved region related to soft CB[8] structures. The corresponding lateral shift of the curves is different since the thickness of the CB[8] deposit is also different. It results to be of 2 nm (short, dashed curve) and 5.5 nm (dashed curve). These values support the identification of the nanostructures on top of the flakes in the Si/MoS_2_/CB[8] sample as CB[8] structures as well as the inhomogeneous character of the CB[8] deposits since their thickness and surface distribution changes with the sample location.

Finally, Fig. 4F shows a 3D representation of one flake in which a very narrow fiber structure is observed. This structure has a height of just 1.3–1.8 nm range, a width in the 15–20 nm range, and a length of at least 900 nm. Due to tip convolution effects, the lateral and height dimensions could be compatible with a fiber partially composed by single CB[8] molecules.

### Electrochemical characterization of the GC/MoS_2_/CB[8] sensor

Cyclic voltammetry experiments were performed with two different proposes: to evaluate the electroactive surface and to investigate the mechanism governing the electrochemical oxidation process.

The evaluation of the electroactive surface was performed with Ru(NH_3_)_6_^2+/3+^ as redox probe as its electron transfer does not depend on an interaction with a functional group or surface place^36^. To this end, cyclic voltammograms of 1 mM Ru(NH_3_)_6_^2+/3+^ in a 1 M KCl solution were recorded at different scan rates for the bare and the modified electrodes. The Randles–Sevcik equation^37^ depicts the relation of the peak current with the electrochemical surface area:

1 $$ {\text{Ip}}\, = \,0.{\text{4463nFAC }}\left( {\frac{nFvD}{{RT}}} \right)^{{{1}/{2}}} . $$

In this equation, Ip is the measured peak current; n is the number of electrons, A the electrochemical surface area, D the diffusion coefficient (7.8 × 10^−6^ cm^2^ s^−1^ for Ru(NH_3_)_6_^2+/3+^ at 20 °C^38^), v the scan rate, F the Faraday constant (9.65 × 10^4^ °C mol^−1^), *R* the gas constant (8.31 J K^−1^ mol), T the temperature (K) and C the concentration of the redox probe (M). From the slope of the corresponding I_pa_ *vs* v^1/2^ plots (Fig. S2A), electroactive surface areas of (0.110 ± 0.008) cm^2^ and (0.19 ± 0.02) cm^2^ were obtained for the bare GC and the GC/MoS_2_/CB[8] electrodes, respectively. These values show that an increase, close to 73%, of the effective area is produced because of the modification.

In addition, cyclic voltammograms of 1.0 mM MEL solutions in 0.2 M phosphate buffer pH 7 at different scan rates ranging from 5 to 350 mV s^−1^ were recorded with the purpose of elucidate the electrochemical mechanism governing the oxidation process. The oxidation wave current of MEL at 0.67 V increases with the scan rate variation. Data were analysed as log Ip *vs.* log V_b_ plot obtaining a linear relation according to log Ip = 0.24 (± 0.02) + 0.489 (± 0.009) log V_b_, r = 0.9993 (see Fig. S2B). The slope value close to 0.5 allows us to conclude that the oxidation takes place mainly through a diffusive process.

Moreover, we carried out EIS experiments to evaluate the influence of the modification in the charge transfer process. MEL was employed as redox probe for a more real evaluation of this interfacial process (Fig. S2C). As shown in the Nyquist plots, an increase in the semicircle at high frequency values is register for the GC/MoS_2_ system respect to the bare electrode revealing an increase in the charge transfer resistance (from 30.000 to 44.000 Ω) as consequence of the modification with the semiconducting material. This resistance increase explains the decrease in the peak current observed for this GC/MoS_2_ system in the differential pulse voltammograms depicted in Fig. 1 (voltammogram b). In contrast, the incorporation of the macrocycle on the electrode surface together with the TMD leads to a decrease in the charge transfer resistance to 25.000 Ω leading to an increase in the peak current as observed in Fig. 1 (voltammogram d).

### Chemical and instrumental variables optimization

All variables involved in the response of MEL were optimized before the evaluation of the corresponding analytical parameters displaying the performance of the sensor.

The influence of the pH of the supporting electrolyte was tested in the 2–11 range employing both, the bare and the CG/MoS_2_/CB[8] electrode. Figure S3 shows the influence of the pH in the peak potential (Ep) value recorded for the oxidation of MEL in each system. As observed, for the CG/MoS_2_/CB[8] electrode (black square dots), the Ep remains constant at acidic pH values. However, it shifted to less positive values when the pH of the supporting electrolyte varies from 5 to 11 according to Ep (V) = 0.89 (± 0.03) − 0.034 (± 0.003) pH; r = 0.992. A similar behaviour was recorded for MEL in the bare GC electrode (red circles dots) although, in this case, Ep shifted to less positive values, from pH = 4. Several conclusions can be drawn from these results. On the one hand, pKa values of 4.0 and 5.0 can be obtained for the “free” and the “complexed” analyte, repectively. Therefore, regarding the acid–base behaviour of MEL, the supramolecular complex formation turns MEL less acidic in comparison with the free MEL. On the other hand, from the slope of the Ep/pH plot, it could be concluded that two-fold electrons than protons are involved in the electrochemical MEL oxidation process with the CG/MoS_2_/CB[8] sensor. All these results are in good agreement with previous studies related to changes in the pKa values in supramolecular systems and with the electrochemical MEL oxidation^28,39^. Finally, as an increase in the oxidation current while increasing the pH value was produced up to pH 7, further experiments were performed using phosphate buffer pH 7 (data not shown).

As a consequence of the negative charge presented in the portals of the CBs, the amount of salts in solution can influence in the complex formation with these supramolecular systems. Figure S4 shows the effect of increasing the electrolyte concentration on the analytical signal of the supramolecular complex when formed on the GC/MoS_2_/CB[8] electrode surface (plot c). The behaviour of the free guest, either with the bare electrode (plot a) or with that modified with MoS_2_ (GC/MoS_2_ system, plot b), are also included for comparison. It is worth noting that upon increasing the phosphate concentration up to 0.2 M, the oxidation response of the free MEL is almost constant (plots a and b), while for the sensor containing CB[8] a great increase in peak current is produced. This behaviour implies that an excess of salt in solution would make easier for MEL to reach the electrode surface and to enter into the CB[8] cavity. This fact is in good agreement with other results previously reported for electrochemical sensors based on CB[n]s^28^. From the results, 0.2 M phosphate buffer at pH 7 was selected as supporting electrolyte for further experiments.

Once the chemical variables were optimized, instrumental parameters involved in the DPV response of MEL were individually evaluated. The pulse amplitude was varied in the 5 to 100 mV range and a current increase was observed up to 60 mV. At greater potential pulse amplitude values, the peak current did not change significantly, and broader peaks were obtained. So, a pulse amplitude of 60 mV was selected looking for to the best compromise between sensitivity and selectivity. The scan rate was varied from 5 to 60 mV/s and was adjusted by the individual studies of the step potential and the interval time. Best results were obtained scanning the potential at 30 mV/s resulting of a step of 15 mV and an interval time of 0.5 s.

### Influence of the MEL concentration: Analytical data

**Figure 5:**
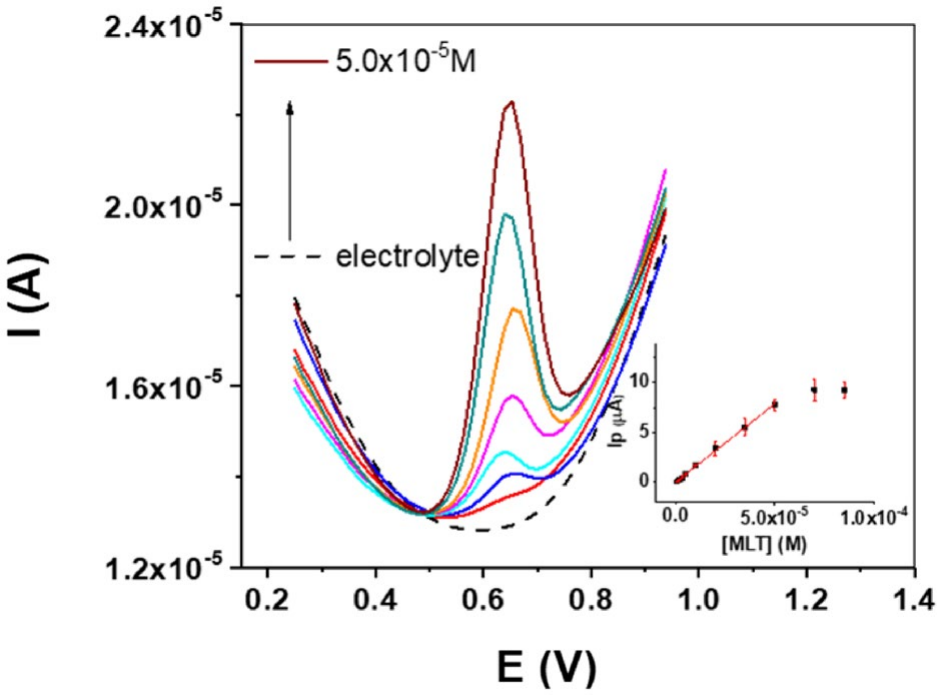
Influence of MEL concentration in the peak current at the GC/MoS_2_/CB[8] sensor. 0.2 M pH = 7.0 phosphate buffer. DPV conditions: scan rate 30 mVs^−1^, pulse amplitude 60 mV and step potential 15 mV.

Once the sensor construction and all chemical and instrumental variables have been optimized towards MEL determination, DPVs of GC/MoS_2_/CB[8] at increasing MEL concentration were recorded (Fig. 5).

The current measured increases linearly as a function of the MEL concentration in the range from 1.25 × 10^–6^ M to 5.00 × 10^–5^ M, according to the equation Ip (µA) = − 0.04 ± 0.02 + (1.58 ± 0.03) × 10^5^[MEL] (M), with a correlation coefficient of 0.997. The sensitivity, calculated as the slope of the calibration curve, was 1.58 × 10^5^ µA × M^−1^. The detection and quantification limits, obtained from the standard deviation of the blank signal and the sensitivity as 3σ/slope and 10σ/slope, were 3.80 × 10^–7^ M and 1.25 × 10^–6^ M, respectively. Table S1 depicts a comparative of the main analytical parameters reported towards MEL determination employing different analytical procedures. Although some examples of extremely low detection limits are reported with sophisticated instrumentation^7^, analytical results obtained in this work are quite similar^15,40,41^ or even better^5,6,11,13^ than other previously reported, without the need to use electrochemical procedures that imply a certain accumulation time of MEL on the electrode surface^9,10^ or a previous activation step^10^, which means an increase in the total analysis time. Moreover, in our case a simple sensor construction is performed using environmentally friendly solvents such as water and ethanol in the working electrode preparation.

The accuracy and precision of the method were evaluated at 5.0 × 10^–6^ M MEL concentration. A relative error of 3.8% was obtained. Precision was estimated in terms of reproducibility with different GC/MoS_2_/CB[8] electrodes (n = 3) obtaining a relative standard deviation value (RSD) of 4.4%. Storage stability was studied by recording weekly DPVs of a GC/MoS_2_/CB[8] sensor. The sensor was kept at 4 °C between measurements. Even after 6 weeks, 85% of the original response was retained, indicating an excellent stability.

### Study of interferences

To evaluate the selectivity of the sensor, dopamine, ascorbic acid and uric acid were assayed as possible interferents in the determination of MEL in biological samples. In addition, pyridoxine (Vit B6) was also evaluated as interferent as this compound is present together with MEL in the pharmaceutical tablets analyzed. Differential pulse voltammograms of 2.0 × 10^–5^ M MEL solutions without and with increasing interferent amounts, were recorded. It was considered that a substance causes interference at a concentration level that led to a change of 10% in the initial analytical signal of MEL. According to this criterium, the maxima concentrations of each interferent that can be present with the analyte without producing interference, were 7.0 × 10^–5^ M, 1.0 × 10^–4^ M and 7.0 × 10^–5^ M for dopamine, ascorbic acid and pyridoxine, respectively. On the other hand, no interference of uric acid was observed in the concentration range assayed (up to fivefold respect to MEL concentration).

### MEL determination in real samples

To test the performance of the sensor in real applications, samples of different nature were subjected to MLT analysis: pharmaceutical formulations (containing 1.95 mg/tablet of MEL), and synthetics human urine and human serum. All procedures for the different sample preparation steps are described in “Procedures”. In the case of pharmaceutical formulations, the results of the standard addition procedure showed that Ip corresponding to the complex oxidation increased with MEL concentration added according to Ip (µA) = (1.2 ± 0.2) + (1.57 ± 0.08) × 10^5^ [MEL] M; r = 0.990. As no significative differences between this slope and that obtained from the calibration equation (1.58 ± 0.03) × 10^5^ µA/M, MEL was quantified by interpolating the corresponding peak current values in the calibration plot. From the results, a MEL amount of 1.92 mg/tablet was obtained. In the case of biological samples (initially spiked at 5.00 × 10^–6^ M MEL final concentration) we found significative differences between the slope obtained with the calibration equation and that obtained from the standard addition procedures (see Table 1). Then, this later procedure was employed for MEL quantification in the case of urine and serum samples. All analysis were carried out in triplicate. Table 1 resumes the results obtained in each case.

**Table 1:** Analytical applications. Results of MEL determination in different samples.

Sample	Procedure	Calibration curve Ip (µA)/[MLT] M	r	Recovery (%) _n=3_	RSD (%)_n=3_
Tablets	External calibration	Ip = (− 0.04 ± 0.02) + (1.58 ± 0.03) × 10^5^ [MEL]	0.997	98	7
Synthetic urine	Standard addition	Ip = (1.5 ± 0.1) + (2.9 ± 0.1) × 10^5^ [MEL]_added_	0.995	102	9
Synthetic serum	Standard addition	Ip = (0.48 ± 0.06) + (1.06 ± 0.06) × 10^5^ [MEL]_added_	0.992	90	9

From these results, we demonstrate that the method proposed is good enough to be applied to MEL determination in real samples of different nature.

## Methods

### Reagents and instrumentation

Molybdenum disulphide (99%, 90 nm powder), cucurbit[8]uril, hexaamin ruthenium (III) and melatonin (99%) were supplied by Sigma Aldrich. Ethanol absolute (EtOH) (99%), acetonitrile, perchloric acid, phosphoric acid and acetic acid were purchased from Scharlau (Barcelona, Spain). All reagents used were of analytical reagent grade. The pharmaceutical tablets employed for the analytical application were obtained from a commercial market. Synthetic serum and synthetic urine samples (both free of melatonin) were supplied by Sigma Aldrich, respectively. Milli-Q water was purified with a Milli Ro Milli Q Plus 185 apparatus from Millipore (Waters, Milford, USA).

Electrochemical measurements were carried out with a µ-Autolab Type III potentiostat employing Gpes software (both from Metrohm Autolab, Utrecht, Netherlands). Electrochemical Impedance Spectroscopy (EIS) was performed with a Frequency Response Analyzer (FRAII) from Eco-Chemie coupled with an Autolab PGSTAT 302N from Eco-Chemie (Utrecht, The Netherlands). A conventional three-electrode system was employed with a glassy carbon electrode (GC; 3 mm in surface diameter either bare or modified) as working electrode. An Ag/AgCl/KCl (3 M) electrode and a coiled platinum wire one were used as the reference and the counter electrode, respectively.

Atomic Force Microscopy characterization was done with a Nanoscope IIIa system (Veeco). The force curves were obtained with an Agilent 5500 PicoPlu6s system (Agilent) and a closed loop and using the same cantilever. The images were analyzed with the Gwyddion free-software package^42^.

### Procedures

#### Nanomaterials synthesis

MoS_2_ nanosheets were obtained following a top-down solvent exfoliation strategy^21^. To this end, 75 mg of MoS_2_ were exfoliated by ultrasonication during 2 h in 10 mL of EtOH/H_2_O (45/55, v/v). The resulting suspension was kept at 4 °C for 24 h and centrifuged at 4000 rpm during 45 min. Next, the supernatant was separated and stored at 4 °C. The CB[8] suspensions (0.3 mM) were prepared in ultrapure water.

#### Electrochemical sensor preparation

Before modification, a commercial GC electrode was polished with 0.3 µm alumina slurry for 1 min, rinsed with ultrapure water and sonicated in ethanol (30 s) and deionized water (30 s) alternatively for three times, and dried under nitrogen flow. Next, the electrode surface was modified by a drop-casting procedure with first, 6 µL of the MoS_2_ nanosheets and, after let it dry at 40 °C, with a second layer of 6 µL of the CB[8] suspension (GC/MoS_2_ /CB[8] system) that was also let drying in the same way. For comparison, the same procedure was carried out only with the MoS_2_ suspension (GC/MoS_2_) or with the CB[8] one (GC/CB[8]).

#### Electrochemical measurements

Differential Pulse Voltammetry (DPV) was employed as electrochemical technique. Voltammograms were performed scanning the potential from 0.25 to 0.95 V, in 0.2 M pH 7 phosphate buffer as supporting electrolyte. The pulse amplitude was 60 mV and the potential was scanned at a scan rate of 30 mV/s as result of applying a step potential of 15 mV and an interval time of 0.5 s. Cyclic Voltammetry experiments were carried out in 0.2 M phosphate buffer pH 7 scanning the potential between 0.25 to 1.00 V at different scan rates. Electrochemical impedance spectroscopy (EIS) measurements were performed at 0.75 V in 0.2 M phosphate buffer solution at pH 7 containing 1.0 mM MEL as electrochemical probe. A sinusoidal potential modulation of ± 10 mV amplitude was applied in the 10^5^ Hz – 10^–2^ Hz frequency range. The experimental data were plotted in the form of Nyquist plots.

#### Atomic force microscopic (AFM) measurements

The AFM measurements were carried out operating in the dynamic mode using silicon cantilevers with a nominal radius of curvature of 8 nm and nominal force constant in the 1–5 N/m range (Bruker). The images were formed by 512 × 512 pixels. The hardest materials (sapphire and MoS_2_) were measured at the end of the experiments to preserve the tip status.

#### Samples preparation

Pharmaceutical formulations: 5 commercial pills (containing 1.95 mg/pill of MEL), with an average weight of 343 ± 2 mg, were crushed in an agate mortar. Next, an accurate amount of about 100 mg of pulverized tablets was dissolved in 0.1 M HClO_4_/20% acetonitrile mixture (c.a. 8 mL) under magnetic stirring, filtered and diluted to 10.00 mL^10^. Next, 125 µL subsamples of the sample solution (non-spiked and spiked at different final MEL concentration levels) were diluted to 5.00 mL with 0.2 M phosphate buffer pH 7, and the corresponding voltammograms were recorded.

Synthetic urine samples were analyzed as follows: 1.0 mL of sample was spiked with MEL and diluted to 10.00 mL with supporting electrolyte before DPV analysis (5.00 × 10^–6^ M MEL final concentration). The standard addition procedure was carried out for MEL determination by spiking this sample at different MEL concentration in the linear range without any additional treatment.

Synthetic serum samples: 100 µL of human serum were diluted with supporting electrolyte, to 5.00 mL after being fortified and filtered through 0.45 µm filter (MEL at 5.00 × 10^–6^ M final concentration). Next MEL was quantified by the standard addition procedure.

## Conclusions

We developed a novel sensor combining MoS_2_ nanosheets and cucurbit[8]uril on the surface of a GC electrode. Optimal performance of the sensor was obtained when a first layer of MoS_2_ dispersed in EtOH/water was deposited on the electrode surface, followed by a second layer of a CB[8] suspension. Images obtained from AFM allowed the identification of plateau–like structures (corresponding to MoS_2_ nanosheets) covered by aggregates of CB[8] adsorbed on its surface. It was proved that the presence of CB[8] altogether with MoS_2_ nanosheets reduces the resistance charge transfer, and also increases the electroactive surface, contributing to the improved performance of the sensor.

Combination of MoS_2_ and the macrocyclic molecular receptor CB[8] enhances the signal of MEL oxidation, which is an unexpected behaviour for the electrochemical oxidation of an inclusion complex, confirming a synergistic effect between both components. Under optimized conditions the sensor showed a linear response with increasing MEL concentrations and was successfully applied on real samples (pharmaceutical formulations, human urine and serum) showing good recovery percentages in all cases. All the data collected in this study open new possibilities on the development of new sensors that combine nanomaterials and macrocyclic molecular receptors, and on their applications.

## Supplementary Information

Supplementary Information.

## Data Availability

The datasets used and/or analysed during the current study available from the corresponding author on reasonable request.
