# Supplementary material for: Electrochemical sensor based on the synergy between Cucurbit[8]uril and 2D-MoS2 for enhanced melatonin quantification
Source: Sci Rep. 2023 Jun 27;13:10378. doi: 10.1038/s41598-023-37401-9 (PMC10300025; doi:10.1038/s41598-023-37401-9)
Supplement: Supplementary file 1 — Supplementary Information. [file 41598_2023_37401_MOESM1_ESM.docx]

**Supplementary information**

**Electrochemical sensor based on the synergy between Cucurbit[8]uril and 2D-MoS_2_ for enhanced melatonin quantification.**

Rut Martínez-Moro^1^, María del Pozo^1^, Luis Vázquez^2^, José A. Martín-Gago^2^, María Dolores Petit-Domínguez^1^, Elena Casero^1*^, Carmen Quintana^1*^.

*^1^Departamento de Química Analítica y Análisis Instrumental. Facultad de Ciencias. c/ Francisco Tomás y Valiente, Nº7. Campus de Excelencia de la Universidad Autónoma de Madrid. 28049 Madrid. Spain*

*^2^Instituto de Ciencia de Materiales de Madrid (CSIC). c/ Sor Juana Inés de la Cruz Nº3. Campus de Excelencia de la Universidad Autónoma de Madrid. 28049 Madrid. Spain*

carmen.quintana@uam.es; Phone: +34 914977626; Fax: +34914974931

elena.casero@uam.es; Phone: +34 914972990; Fax: +34914974931

Figure S1

**Figure S1.** Oxidation current obtained from 1 × 10^-5^ M MEL solutions with increasing CB[8] ratios with a GC electrode in 0.1 M pH=7.0 phosphate buffer solution.

Figure S2

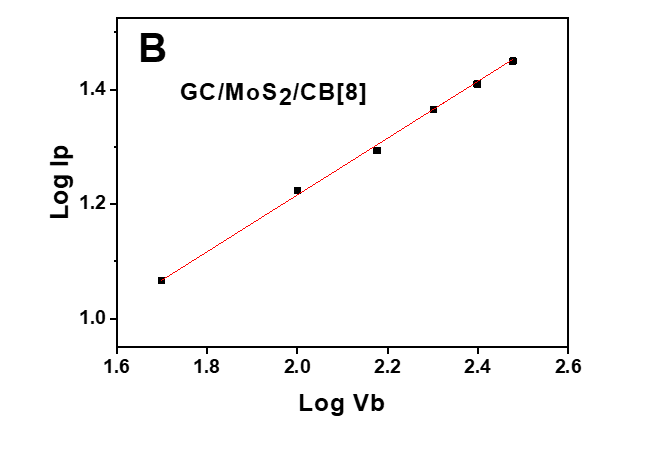


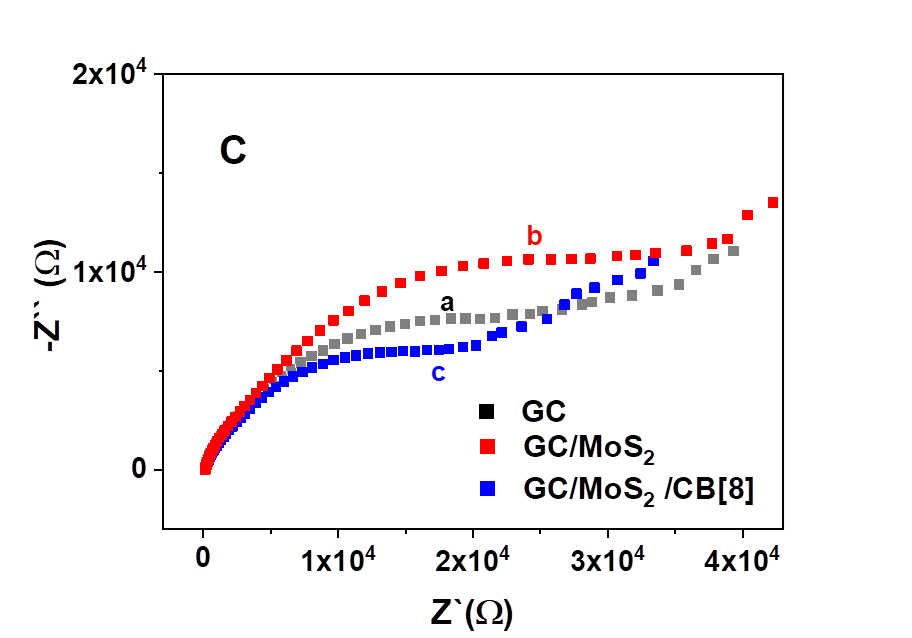


**Figure S2**. *Electrochemical characterization of the GC/MoS_2_/CB[8] sensor:* A) Variation of anodic Ip current with V_b_ ^1/2^obtained from 1 mM Ru(NH_3_)_6_^2+/3+^ in 1 M KCl solutions. B) Log Ip v.s. log V_b_ plot obtained from the differential pulse voltammograms of 1.0 mM MEL solutions in 0.2 M phosphate buffer pH 7 at different scan rates. C) Nyquist plots of 1.0 mM MEL in 0.2 M phosphate buffer pH 7 obtained with the bare GC electrode (a), GC electrode modified with MoS_2_ (b) and GC electrode modified with MoS_2_ /CB[8] (c).

Figure S3


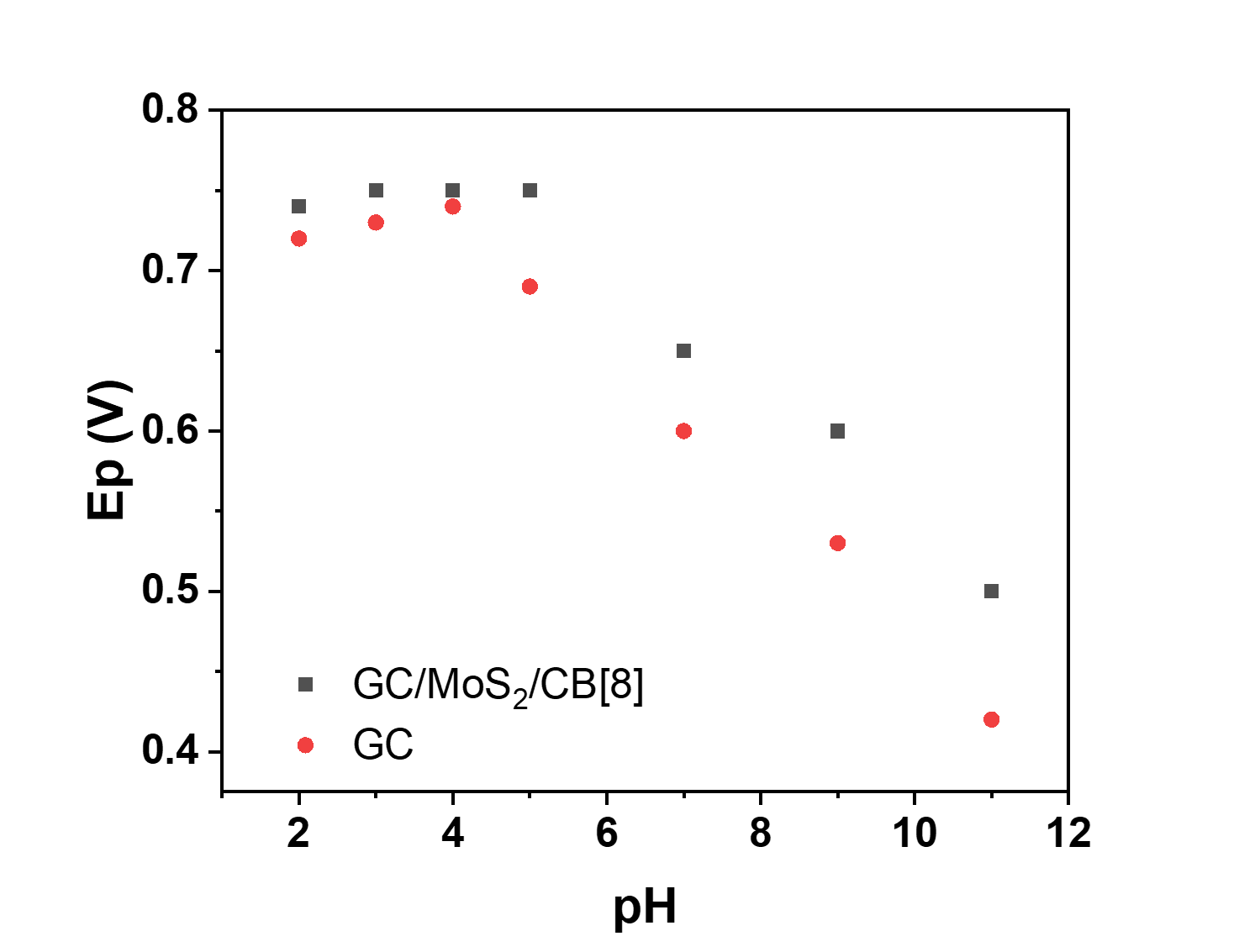


**Figure S3**. Influence of pH in the peak potential for GC and GC/MoS_2_/CB[8] electrodes. 10^-5^ M MEL in 0.1 M pH=7.0 phosphate buffer. DPV conditions: scan rate 30 mVs^-1^, pulse amplitude 60 mV and step potential 15 mV.

Figure S4

**Figure S4.** Influence of the supporting electrolyte concentration in the MEL oxidation with a) GC electrode, b) GC/MoS_2_ electrode, c) GC/MoS_2_/CB[8] system. [MEL] = 10^-5^ M. DPV conditions: scan rate 30 mVs^-1^, pulse amplitude 60 mV and step potential 15 mV.

**Table S1:** Comparison of relevant analytical parameters towards Mel analysis with different analytical procedures

| **Analytical Method** | **LOD (M)** | **Linear Range** | **Reference** |
| --- | --- | --- | --- |
| LC-MS/MS | 9.6 – 52.9 pg/g | 2.5·10^-10^ – 8.6·10^-8^ | 7 |
| OSWSV. Activated GCE | 5.0·10^-8^ | 8.0·10^-7^ – 1.0·10^-5^ | 9 |
| FPA (GPH-CSPE). | 8.7·10^−7^ | Not specified | 11 |
| CE-ED | 1.3·10^-6^ | 2.5·10^-6^ – 1.0·10^-3^ | 5 |
| HPLC – F | 2.6 ·10^-4^ | 2.2x10^-6^ -8.6·10^-4^ | 6 |
| SWV (S-AIPE) | 4.9· 10^-7^ | 1.0 ·10^-5^– 1.0·10^-4^ | 40 |
| DPV  GON-SPE | 1.1·10^-6^ | 5·10^-6^ – 3·10^-3^ | 13 |
| SWV  AHNSA:PdNPs:ErGO/GCE | 0.9·10^-7^ | 5·10^-6^ – 1·10^-4^ | 15 |
| SWV  RGO-Cu_0.5_Co_0.5_Fe_2_ O_4_ /Pt | 1.72·10^-7^ | 2.0·10^-7^ - 2.0·10^-5^ | 41 |
| DPV  GCE/MoS_2_/CB[8] | 3.8·10^-7^ | 1.0 10^-6^ – 50.0 ·10^-6^ | This work |

LC-MS/MS: Liquid chromatography Tandem Mass Spectrometry; OSWSV: Osteryoung square-wave stripping voltammetry; GCE: Glassy Carbon electrode; SWV: square wave voltammetry; GPH-CSPE:graphene-carbon screen-printed electrode; FPA: fixed-potential amperometry; CE-ED: capillary electrophoresis with electrochemical detection; DPV: differential pulse voltammetry. GON-SPE: graphene oxide nanoribbons on screen-printed electrodes, S-AIPE: disposable self-adhesive inked paper electrode AHNSA:PdNPs:ErGO/GCE composite on glassy carbon electrode: AHNSA: 4-amino-3-hydroxy-1-naphthalene sulfonic acid ErGO : Electrochemically reduced graphene, PdNPs: palladium nanoparticles
